# The rediscovery of *Uraria lacei* Craib (Leguminosae) after 67 years from India

**DOI:** 10.3897/phytokeys.160.54237

**Published:** 2020-09-08

**Authors:** Jahnabi Gogoi, Tikam Singh Rana

**Affiliations:** 1 Academy of Scientific and Innovative Research (AcSIR), Ghaziabad-201002, India Academy of Scientific and Innovative Research Lucknow India; 2 Molecular Systematics Laboratory, Plant Diversity, Systematics and Herbarium Division, CSIR-National Botanical Research Institute, Lucknow-226001, India National Botanical Research Institute Lucknow India

**Keywords:** Biodiversity hotspot, Fabaceae, Manipur, rediscovery, *
Uraria
*

## Abstract

Manipur is one of the biodiversity-rich states in the North-Eastern region of India, and it is also part of the Indo-Burma biodiversity hotspot with rich plant diversity and endemism. Recent field exploration in the area has resulted in the rediscovery of *Uraria lacei* Craib after 67 years from its last collection in 1952. The rediscovery of this beautiful species fills a gap in the current distribution knowledge and should pave the way for its immediate conservation and propagation.

## Introduction

The genus *Uraria* Desv. (Fabaceae-Papilionoideae-Desmodieae) contains about 20 species distributed in tropical Africa, South East Asia and Australia (Ohashi et al. 2006). In India, the genus has eight to 12 species predominantly found in tropical and sub-tropical regions (Baker 1879, Sanjappa 1992, Gaur 1999, Kirtikar and Basu 2001). *Uraria lacei* Craib is a beautiful species with dark blue inflorescence and is distributed in India, China, Laos, Myanmar, Thailand and Vietnam. The panicle of *U. lacei* is similar to that of *U. oblonga* (Wall. ex Benth.) H. Ohashi & K. Ohashi (Ohashi et al. 2018). In India, *U. lacei* is reported from the states of Nagaland, Manipur, Mizoram and Bihar (Sanjappa 1992). However, published literature and herbarium data suggests that, although the species was mentioned in the recent floras of Manipur (Singh et al. 2000) and Mizoram (Singh et al. 2002), these records were based on older collections. During a field study in November 2019 by the first author to North East India, the species was collected in flowering and fruiting stages from a hillslope at the only floating National Park (Keibul Lamjao National Park, Bishnupur, Manipur) in the world. After compiling all the data available in the public domain and specimens in different herbaria, it was observed that the last collection was from 11 September 1952 (*D.B. Deb 585*, CAL!). In the past 67 years, the species was not recollected from its occurrence in India, therefore, this certainly raises questions about its current status, conservation and existence in nature. Detailed description, taxonomic notes and colour photographs of *U. lacei* are provided here.

## Methods

All the published literature were scrutinized for the probable localities or distribution of *Uraria* spp. in India. With that distribution data, a field survey was conducted during October–November 2019 in the states of Assam, Nagaland, Manipur and Meghalaya of India to collect plants of *Uraria* spp. Fresh specimens of *U. lacei* were collected in flowering and fruiting stages from Manipur. The flowering twigs were packed in airtight polybags, flowers and fruits were separately collected in collection tubes containing 70% ethanol for further studies. Field notes recorded included habit, habitat, number of individuals in the population, geo-coordinates, and elevation data. In transit camp, the specimens were pressed and dried on blotting sheets. Upon reaching the institute, they were processed in the herbarium following standard herbarium procedures (Jain and Rao 1977). All the collected samples were dissected and were examined under stereo microscope (LEICA S8 APO, Wetzler, Germany) and described. Taxonomic literature and protologues were studied and compared for identification (Hasskarl 1844; Clarke 1889; Lecomte 1920; Haines 1921; Deb 1961; Thuan et al. 1987; Sanjappa 1992; Sha 1994; Singh et al. 2000; Singh et al. 2002; Kumar and Sane 2003; Ohashi et al. 2006; Puhua et al. 2010).

Herbarium sheets were consulted which are available in GBIF as well as various Indian (AHMA, ARUN, APF, ASSAM, BSA, BSD, BSI, BSID, CAL, DD, FRC, LWG, MH and TBGT) and foreign digital herbaria (A, B, BM, BO, E, H, K, L, MO, NY, P and US) (acronym following Thiers 2018). The specimens of *U. lacei* were found in DD, CAL, K, P, E, MO, L, A and US. The collected voucher specimens have been deposited at LWG. The distribution map was prepared using QGIS 3.8.0- Zanzibar.

## Taxonomy

### Uraria lacei

Taxon classificationPlantaeFabalesLeguminosae

Craib, Bull. Misc. Inform. Kew 1910(8): 276 (1910)

26F1E3F6-41A7-528F-AAEA-9F5DEADDF96B

Figs 1 , 2

#### Type.

Myanmar. Maymyo Plateau, 3500 ft, 12 Oct 1908, *J.H. Lace 4325* (lectotype, designated by Thuan et al. 1987, pg. 112: K! [K000858898]; isolectotypes: CAL! E! [E00301323, E00813650])

#### Synonyms.

*U. paniculata* C.B. Clarke, J. Linn. Soc. Bot. 25: 15, tab. 4. 1889. *nom. illeg*. Type: India. Kohima, 3000 ft, 19 Oct 1885, *C.B. Clarke 40924* (K!). *U. clarkei* Gagnepain in Lecomte, Fl. Gen. Indoch. 2: 542. 1920. Type: Vietnam. Tonkin: Plateau de Kiendi, dans les paturages, 7 Oct 1891, *B. Balansa 4430* (Holotype: P [P02142551]). *U. pulchra* Haines, Bull. Misc. Inform. Kew 1921 (8): 308. 1921. Type: India. Bihar, Someshwar Hills, *H.H. Haines 3962* (ABD!). *U. guangxiensis* W.L. Sha in Guihaia 14: 23. 1994. Type: China. Guangxi, Nandan Xian, Yueli, 20 Sept 1977, Exped. *Nandan 4–5–073*; (Lectotype, designated by Ohashi et al. 2006, pg. 344: Herb. Guangxi Research Centre of Natural Materia Medica).

**Figure 1.**
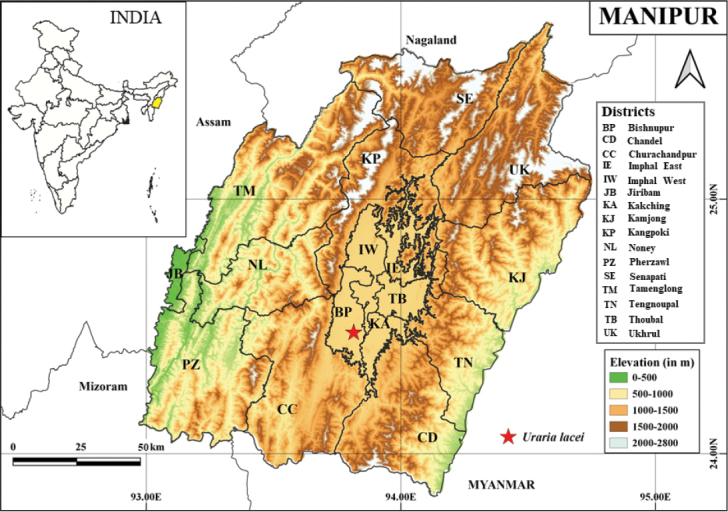
Map of Manipur showing the locality where *Uraria lacei* was rediscovered.

#### Description.

Shrubs up to 3 m height. Roots taproot with lateral roots. Stems erect, solid, strong, striate, 0.5–0.9 cm wide with ferruginous hooked hairs (0.018–0.099 cm long) and straight hairs (0.008–0.045 cm long), internodes 0.7–3.0 cm long. Leaves trifoliolate, rarely 4-foliolate, 8.0–21.5 × 7–17 cm; petiole terete, 1.5–5.0 × 0.2 cm, scabrous, up to 0.1 cm long hairs; rachis 1.0–2.5 × 0.1–0.2 cm; petiolule densely scabrous, 0.2–0.4 cm long; terminal leaflets 3.7–13.2 × 2.1–7.5 cm, lateral leaflets smaller than terminal, 4.0–10.5 × 1.4–4.1 cm, leaflets elongated ovate, obtuse at both ends, margin entire to crenate, apex mucronate; lateral veins 9–14 pairs, up to margin, with dense brown hairs underneath; midrib protruding underneath, with both dense long straight and hooked brown hairs; leaf blade adaxial (upper) surface pilose with both eglandular straight and hooked hairs, few scattered glandular straight hairs, blade with granular deposition; leaf blade abaxial (lower) surface tomentose soft hairs, shines white in sunlight. Stipules 2, not covering whole of stem width, triangular, caudate, 0.9–1.5 × 0.3–0.6 cm with scabrous hooked and straight hairs; stipels elongated triangular, 0.3–0.5 cm long with scattered scabrous hairs. Inflorescence a very lax panicle, 17–40 × 6–24 cm, ferruginous hairy, young panicle with many bracts and short secondary rachis, mature panicle with deciduous bracts and longer secondary rachis, panicle terminal, sometimes axillary; secondary rachis green, with yellowish-white glandular hairs and short hooked brown hairs; flowers in pairs; pedicels 0.8–1.0 cm long, violet-purple, minutely bent towards calyx, with short hooked white hairs (0.017 cm long). Calyx valvate, 0.3–0.5 cm long, violet-purple, sepals 5, persistent, upper two lobes completely joined together except slightly at the tooth, lower three free at teeth, joined at tube, both lobes almost of equal length, lobes abaxially glandular hairy. Corolla dark blue, petals 5, standard suborbicular, 0.87–0.88 × 0.97–1.02 cm, with two white spots adaxially towards base; wings dark blue, purple, towards base white, 0.81–0.86 × 0.38–0.48 cm, auricle slightly drooped, up to 0.12 cm long; keel-petals 0.99–1.04 × 0.44–0.51 cm, auricle minute, up to 0.04 cm long. Androecium 9 + 1, filament 1.04–1.08 cm long, filament sheath 0.83–0.88 cm × 0.14–0.15 cm, filament tips 0.05–0.14 cm long; anthers 0.07–0.08 × 0.04–0.06 cm. Gynoecium 1.15–1.27 × 0.05 cm, ovary 0.51–0.55 cm long, with 4–8 ovules, slightly appressed hairy, style ca. 0.62 cm long, bent. Pods coiled, 4–8 articles, 0.7–1.2 × 0.3–0.4 cm, green to brownish, with long glandular hairs (0.03–0.06 cm) and minute eglandular hooked (0.008–0.019 cm long) hairs on the joints; seeds yellowish, 0.23–0.25 × 0.18–0.20 cm.

**Figure 2.**
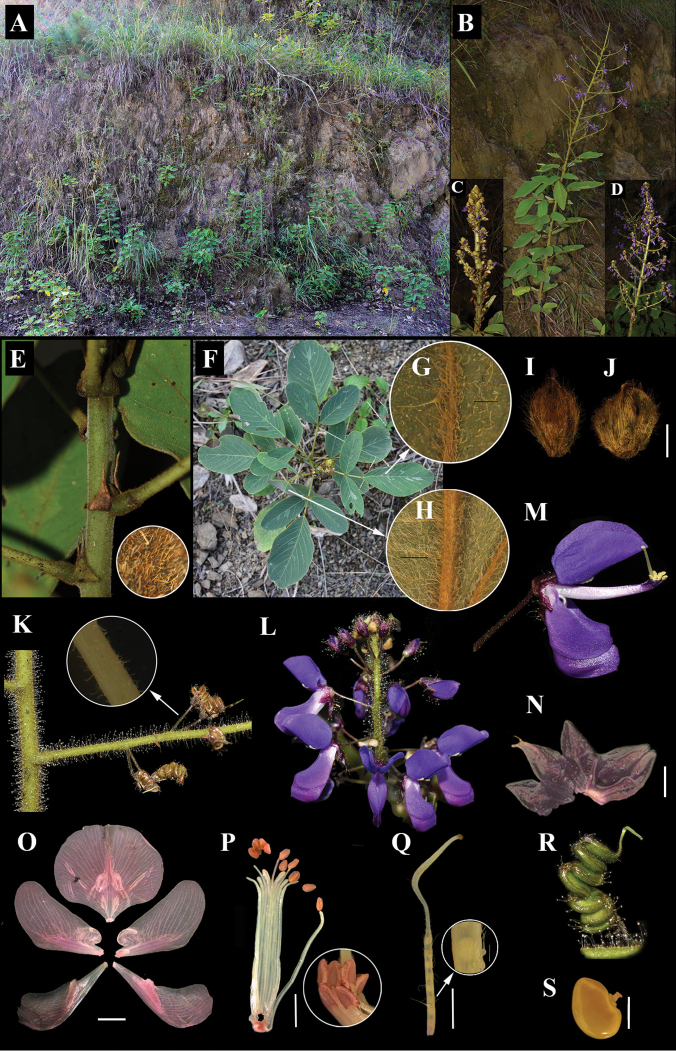
The first photographs of *Uraria lacei* Craib **A** habitat **B** habit **C** young panicle **D** mature panicle **E** stipules and magnified stem hairs **F** leaflets **G** upper surface of leaf **H** lower surface of leaf **I** abaxial surface of bract **J** adaxial surface of bract **K** rachis and fruit position with magnified hairs of pedicel **L** flower position on the panicle **M** single flower **N** calyx (Safranin stained) **O** petals–standard, wings, keel (Safranin stained) **P** androecium with magnified anthers (Safranin stained) **Q** gynoecium with magnified hairs **R** single pod **S** single seed. Scale bars: 2 mm (**J, N–Q**); 500 µm (**S**).

#### Phenology.

Flowering from October to November, fruiting from November to December.

#### Specimens examined.

China. Yunnan: No locality, No altitude, 1897, *A. Henry 9144* (CAL); Szemoa, *A. Henry 9144A* (US02055945); Mengtze, *A. Henry 9144C* (MO-2331548, A00234907, A00234912); *A. Henry 9144* (US-02055943, 02055944); Puerh, *A. Henry 9144C* (K000858912); Southern Yunnan, Between Muang Hai and Keng Hung, 15–17 Feb 1922, *J.F. Rock 2492a* (US-02055941); West of Talifu, Mekong watershed, en route to Youngchang and Tengyueh, Sept.-Oct. 1922, *J.F. Rock 6615, 6585* (US-02055940, 02055942); India. Manipur: Manipur, Laimatak, 3–4000 ft, Nov 1907, *A. Meebold 6245* (CAL); Myring Naga Hills, 5000 ft, Dec 1907, *A. Meebold 9263* (CAL); Litan, 3000 ft, 12 Nov 1944, *N.L. Bor 18132* (CAL); Palel, 3000 ft, 13 Nov 1945, *A.H. Bullock 793* (L0477544); Karong, 3500 ft, 26 Sept 1950, *Walter N. Koelz 26287* (L0477545); Imphal, 11 Sept 1952, *D.B. Deb 585* (CAL); Bishnupur district, Keibul Lamjao National Park, 24.475687°N, 93.814391°E, 773 m, 17 Nov 2019, *Jahnabi Gogoi 327778, 327779* (LWG-106051, 106052, 106050, 106053); 24.475614°N, 93.814607°E, 758 m, *Jahnabi Gogoi 327780* (LWG-106054, 106054); 24.475590°N, 93.814555°E, 755 m, *Jahnabi Gogoi 327781* (LWG-106056); Nagaland: Kohima, 3000 ft, 19 Oct 1885, *C.B. Clarke 40924*; *S.N. Bal 513* (CAL); Naga Hills, Assam, 1935, *N.L. Bor 32* (DD); Myanmar. Maymyo Plateau, 3500 ft, 31 Oct 1911, *J.H. Lace 5512* (DD, E00899264); Maymyo plateau, 3500 ft, 5 Oct 1912, *J.H. Lace 4325/ 5512*? (E00899265); Shwebo District, Kanza Laga Reserve, near Maukaw Forest Rest House, under 1000 ft, 16 Nov 1917, *C. Gilbert Rogers 670* (CAL, DD); Myit Kyi na District, Ka dw nan Pa law, 9 Nov 1930, *Maung Ba Pe 11834* (CAL, DD); Vietnam: Plateau de kiendi, dans les paturges, 7 Oct 1891, *B. Balansa 4430* (P02142551); N. du Tonkin, 900 m, 31 Dec 1937, *M. Poilane 26958* (P02996196, P03089173).

#### Conservation status.

Based on the available literature in the public domain, and our recent field studies, we suggest that *U. lacei* can be provisionally considered under the ‘Data deficient’ category of IUCN (IUCN 2019). However, a further assessment of the threat operating on the species in question needs to be done as per IUCN guidelines.

## Discussion

*Uraria lacei* was first collected by C.B. Clarke on 19 Oct 1885 from Kohima, Nagaland, India. He published the novelty as *U. paniculata* C.B. Clarke in 1890, but was unaware of the fact that the same name exists for a different type *U. paniculata* Hassk. in 1844, thus making it as a later homonym. Gagnepain, in 1920, realised this and renamed it *U. clarkei* Gagnepain, giving credit to C.B. Clarke. In the meantime, Craib (1910) already published it as *U. lacei* Craib, thereby it became the accepted name with priority. *Uraria lacei* was named after the collector of the type specimen, John Henry Lace, a famous botanist and forester in India, Myanmar, Pakistan, etc. His collection period was from 1889 to 1912. There are many species named after him including *Styrax lacei* W.W. Smith (Styracaceae), *Parastyrax lacei* (W. W. Smith) W. W. Smith (Styracaceae), *Derris lacei* Dunn (Fabaceae), and *Euphorbia lacei* Craib (Euphorbiaceae).

*Uraria lacei* is completely different from other species of *Uraria* in its inflorescence. Although the panicle resembles *U. oblonga* (Wall. Ex Benth.) H. Ohashi & K. Ohashi, it differs in having trifoliolate leaves rather than the unifoliolate leaves of the latter. The field observation revealed that the rediscovered population had about 20 individual plants within 2 m^2^ area on the slope of a small hill at Keibul Lamjao National Park, Bishnupur. There were both young saplings as well as mature 2–3 m tall plants. Most of the plants were in flowering and fruiting condition. The soil was sliding due to clearance for road and mostly consisted of small pieces of rocks. The plants were growing with grasses and pines.

**Figure 3.**
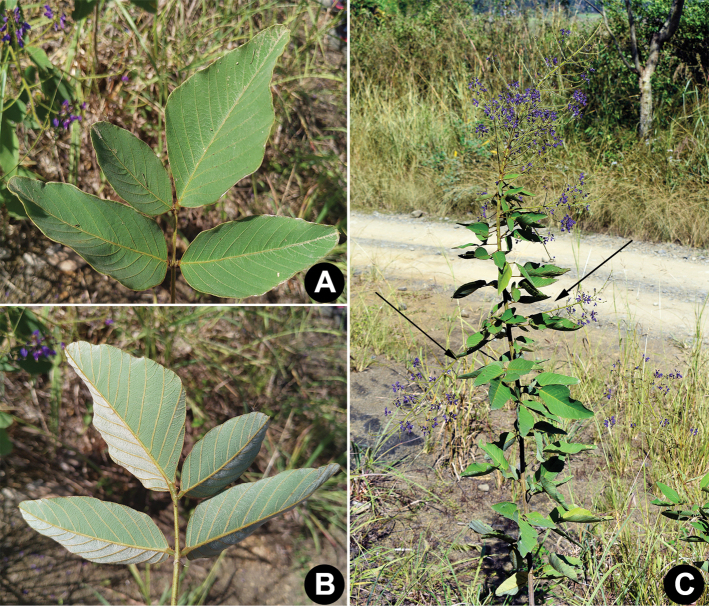
*Uraria lacei* Craib rare 4-foliolate leaflet **A** adaxial surface **B** abaxial surface **C** plant bearing axillary inflorescence (signified by arrows).

While going through the protologue and various literature, it was observed that the species is uniformly described to have terminal inflorescence. However, we observed both terminal as well as axillary panicle during the field survey (Fig. 3). The protologue also suggested the pods to be “fere glabrum” (i.e. almost smooth), which can also mean there might be scarce minute hairs that are not noticeable. On the plants that we collected, the glandular hairs were clearly visible with naked eyes, but were not very dense. Unlike the dense glandular haired pedicel described by Ohashi et al. 2006 from China, the specimens collected were observed to have minute hooked hairs on its pedicels. Thuan et al. (1987) described the calyx as glabrous while Ohashi et al. (2006) described the calyx as densely glandular hairy. Our specimens were neither glabrous nor densely glandular hairy, but were scarcely glandular hairy. As observed in the field, the plants rarely have 4-foliolate leaflets (Fig. 3).

As the plant has beautiful inflorescence and foliage, it would therefore be well-suited for domestication as an ornamental plant. Keibul Lamjao National Park, Manipur (India) is itself a protected area but anthropogenic activities like tourism is allowed in the buffer zone, therefore the vulnerability of *U. lacei* still cannot be ruled out. We could not locate any other population nearby to the present location and, given its rarity, there is an urgent need to conserve the population of this species in its present locale. Furthermore, species specific habitats need to be identified using ecological niche modelling (ENM) tools and saplings multiplied using both macro as well micro-propagation techniques, should be planted in the specific habitats to ensure the *in-situ* conservation of the species.

## Supplementary Material

XML Treatment for Uraria lacei
